# Veterinary Education

**Published:** 1894-09

**Authors:** 


### VETERINARY EDUCATION.

This much discussed topic is taking very difinite form, and within the next few years it is certain that those schools who do not come up to the standard demanded by all fair-minded practitioners, as absolutely necessary, will become extinct. At each and every funeral the list of mourners will be confined to those whose financial standing falls with the decrease in the number of students.

Spasmodic attempts, as of yore, to establish short-term schools will undoubtedly be made by those illiterate or unprincipled individuals who can only see fame and fortune through the illuminated *handbills* of *our school*, which promises much, at very little expense of time, talent or money.

The success of these spasmodic attempts must become less as our ranks become filled with men of broader views and higher ideals. The title of *professor* should be sacred, and should be held only by the best in our profession.

The only reason for the establishment of new institutions should be, except for geographical considerations, the ability to do that which schools already in existence find it impossible to do.

